# Bibliometric analysis of global research trends in adeno-associated virus vector for gene therapy (1991-2022)

**DOI:** 10.3389/fcimb.2023.1301915

**Published:** 2023-12-08

**Authors:** Fengqi Jiang, Chuanhe Zhang, Weina Liu, Fangyuan Liu, Haiyan Huang, Yao Tan, Bo Qin

**Affiliations:** ^1^ Jinan University, Guangzhou, China; ^2^ Shenzhen Aier Eye Hospital, Aier Eye Hospital, Jinan University, Shenzhen, China; ^3^ Shenzhen Aier Ophthalmic Technology Institute, Shenzhen, China

**Keywords:** adeno-associated virus, gene therapy, bibliometric, research trends, inherited diseases

## Abstract

**Background:**

Gene therapy involves introducing and editing foreign genes in the body to treat and prevent genetic diseases. Adeno-associated virus (AAV) vector has become a widely used tool in gene therapy due to its high safety and transfection efficiency.

**Methods:**

This study employs bibliometric analysis to explore the foundation and current state of AAV vector application in gene therapy research. A total of 6,069 publications from 1991 to 2022 were analyzed, retrieved from the Science Citation Index Expanded (SCI-E) within the Web of Science Core Collection (WoSCC) of Clarivate Analytics. Institutions, authors, journals, references, and keywords were analyzed and visualized by using VOSviewer and CiteSpace. The R language and Microsoft Excel 365 were used for statistical analyses.

**Results:**

The global literature on AAV vector and gene therapy exhibited consistent growth, with the United States leading in productivity, contributing 3,868 papers and obtaining the highest H-index. Noteworthy authors like Wilson JM, Samulski RJ, Hauswirth WW, and Mingozzi F were among the top 10 most productive and co-cited authors. The journal “Human Gene Therapy” published the most papers (n = 485) on AAV vector and gene therapy. Current research focuses on “gene editing,” “gene structure,” “CRISPR,” and “AAV gene therapy for specific hereditary diseases.”

**Conclusion:**

The application of AAV vector in gene therapy has shown continuous growth, fostering international cooperation among countries and institutions. The intersection of gene editing, gene structure, CRISPR, and AAV gene therapy for specific hereditary diseases and AAV vector represents a prominent and prioritized focus in contemporary gene therapy research. This study provides valuable insights into the trends and characteristics of AAV gene therapy research, facilitating further advancements in the field.

## 1 Introduction

Gene therapy is a therapeutic approach aimed at introducing foreign genetic material into target cells using non-viral or viral vectors to correct or supplement defective or abnormal genes, thereby treating or preventing inherited diseases (High and Roncarolo, 2019). This innovative therapy can be accomplished through *in vivo* and ex vivo pathways (Dunbar et al., 2018). The ex vivo strategy involves collecting either autologous or allogeneic cells from the human body, followed by the introduction of the foreign gene modification and subsequent cell amplification. These modified cells are then transfused back into the patient’s body. Conversely, in *in vivo* gene therapy, exogenous genetic material is directly introduced into the patient’s target cells by assembling it within a vector. Gene therapy has demonstrated considerable efficacy in treating various hereditary diseases, including blood system disorders such as hemophilia (Miesbach et al., 2018; Perrin et al., 2019), nervous system ailments like Parkinson’s disease (Deverman et al., 2018), retinal genetic diseases affecting the eyes (Bainbridge et al., 2015; Moore et al., 2018; Rodrigues et al., 2018), muscular diseases such as Duchenne muscular dystrophy (Duan, 2018), and cardiac diseases (Ishikawa et al., 2018) through *in vivo* targeted gene therapy.

The successful transduction of genes necessitates the use of specific vectors and techniques to ensure sustained and stable expression of normal genes within the patient’s body. Among the viral vectors employed in gene therapy, the Adeno-Associated Virus (AAV) vector has gained widespread acceptance due to its remarkable features, including high transfection efficiency (Colella et al., 2018) and a favorable safety profile (Le Meur et al., 2018). Notably, the AAV vector elicits only minimal and relatively mild immune responses (Bessis et al., 2004). Remarkable advancements have been made in utilizing AAV vectors for therapeutic purposes. For instance, the treatment of Leber congenital amaurosis with the RPE-specific 65KDa gene (RPE65) carried by the AAV vector has demonstrated significant improvements in visual function (Maguire et al., 2008; Le Meur et al., 2018). Furthermore, clinical trials involving AAV vector gene therapy in patients with hemophilia A and B have exhibited augmented activity of clotting factors in the blood (Nathwani et al., 2014; George et al., 2017; Rangarajan et al., 2017). Beyond these achievements, AAV has exhibited effectiveness in treating other diseases, including cardiac, muscular, and neurological disorders (Chien et al., 2017; Mendell et al., 2017; Bass-Stringer et al., 2018). Its versatility and promising outcomes have positioned the AAV vector as a prominent tool in the arsenal of gene therapy strategies.

Currently, bibliometrics serves as a valuable method for systematically analyzing and summarizing research fields. By employing bibliometric techniques, one can ascertain the contributions and collaborations among authors, institutions, countries, and journals within a specific domain, thus shedding light on the developmental trends in scientific research (Ma et al., 2021). Notably, the field of gene therapy has experienced a surge in research endeavors as science and technology continue to advance. Despite the growing body of research on gene therapy, there remains a dearth of systematic evaluations concerning the application of Adeno-Associated Virus (AAV) in gene therapy. To address this knowledge gap, the present study aims to comprehensively summarize and visually evaluate AAV gene therapy publications using the Web of Science as the primary data source. To facilitate this analysis, the study will leverage CiteSpace (Synnestvedt et al., 2005) and VOSviewer (Van Eck and Waltman, 2010) software, allowing for an in-depth exploration of cutting-edge research and emerging trends within this specific area. By employing robust bibliometric methodologies and advanced visualization tools, this study seeks to provide valuable insights into the state of AAV gene therapy research, offering researchers, policymakers, and healthcare practitioners a comprehensive understanding of the field’s current landscape and its potential future directions.

## 2 Methods

### 2.1 Data retrieval and search strategy

The Web of Science is internationally recognized as one of the most authoritative scientific literature retrieval tools. It encompasses a vast collection of academic journals, books, and proceedings spanning the sciences, social sciences, and arts and humanities. In this bibliometric study, data were sourced from the Science Citation Index Expanded (SCI-E) within the Web of Science Core Collection (WoSCC) of Clarivate Analytics provided by Clarivate Analytics. In addition, using prospectively designed search and selection criteria to ensure quality of retrieval, inclusion criteria following were searched to identify data detailing Genetic Therapy and Adeno-Associated Virus. The search strategy focused on subject term limiting was set to TS=(“Genetic Therapy” OR “Genetic Therapies” OR “Therapies, Genetic” OR “Therapy, Genetic” OR “Therapy, DNA” OR “DNA Therapy” OR “Genetic Therapy, Somatic” OR “Genetic Therapies, Somatic” OR “Somatic Genetic Therapies” OR “Somatic Genetic Therapy” OR “Therapies, Somatic Genetic” OR “Therapy, Somatic Genetic” OR “Genetic Therapy, Gametic” OR “Gametic Genetic Therapies” OR “Gametic Genetic Therapy” OR “Genetic Therapies, Gametic” OR “Therapies, Gametic Genetic” OR “Therapy, Gametic Genetic” OR “Gene Therapy” OR “Therapy, Gene” OR “Gene Therapy, Somatic” OR “Somatic Gene Therapy” OR “Therapy, Somatic Gene”) AND TS=(“AAV” OR “Adeno-Associated Virus” OR “Adeno Associated Virus” OR “Virus, Adeno-Associated” OR “Virus, Adeno Associated” OR “Viruses, Adeno-Associated” OR “Viruses, Adeno Associated” OR “Adeno-Associated Viruses” OR “Adeno Associated Viruses”) that were confirmed from Medical Subject Headings (MeSH) of pubmed for searching literature in wide scope of coverage. (2) For reflecting the research of the subject progress, the retrieval time span was from 1991 to 2022. (3) The setting of limiting condition was only English language.

The exclusion criteria for this study are as follows: (1) Documents that are not related to “Genetic therapies and Adeno-Associated Virus”. (2) Documents classified as meeting abstracts, proceeding retracted publications, reprints, or retractions. (3) Documents not in the English language. Two researchers independently conducted the literature retrieval from the Web of Science Core Collection database, focusing on the relevant topic and target date. The “full records and cited references” function was utilized to export and store the document data as download_txt format for further analysis. The selected parameters from the included literature were subjected to analysis. In case of any divergences encountered during the process, discussions and negotiations between the two researchers were employed to reach an agreement. If a consensus could not be reached, a third party, experienced experts in this field, was consulted to make the final decision. Since this study solely used data from public databases and did not involve human or animal subjects, ethical approval was not required.

### 2.2 Data screening and analysis

**Figure 1:**
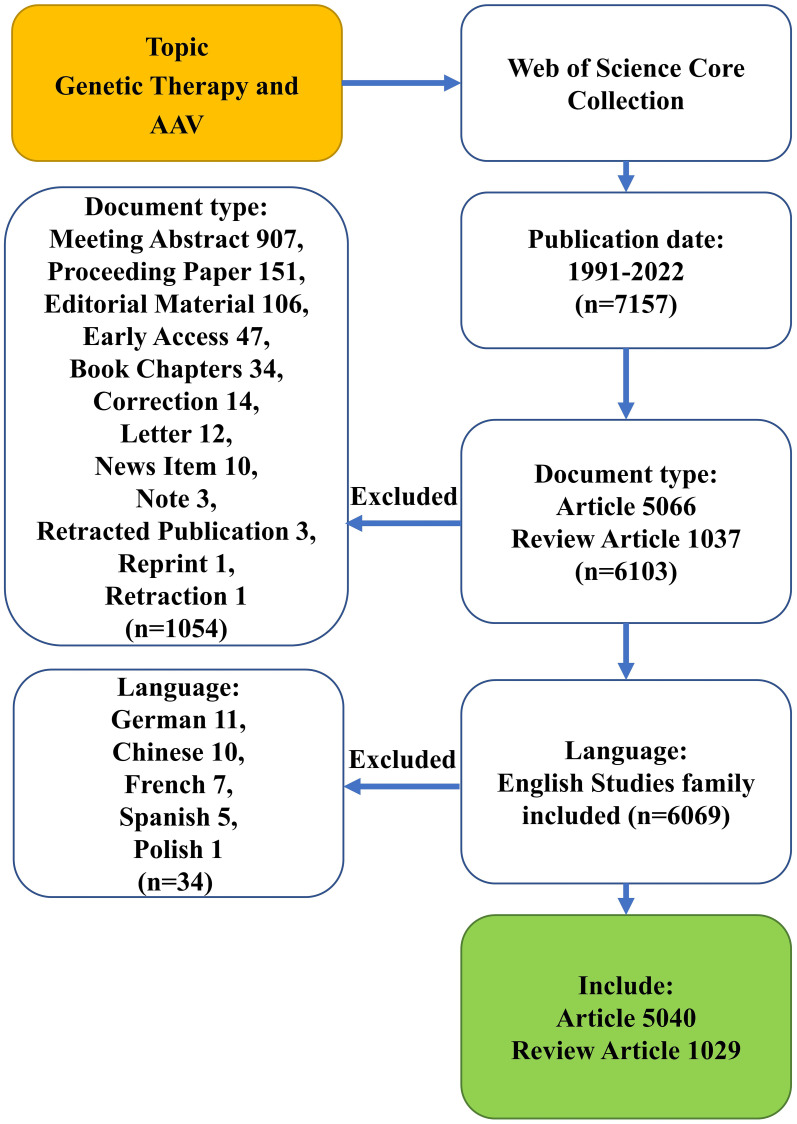
Flowchart of the screening process.

The workflow of this study, depicted in **Figure 1**, involved exporting a total of 6,069 papers from the Web of Science database, comprising 5,040 “articles” and 1,029 “reviews” on Genetic therapies and Adeno-Associated Virus. Three authors extracted relevant parameters and references from the retrieved documents. These parameters included information about authors, such as the number of articles published, author distribution, and cooperation networks. The study also analyzed the contributions of countries and research institutions, the distribution of articles among countries and institutions, and the accumulated number of articles from each institution. Additionally, the study examined the number of articles published each year, document citations, and the total number of document co-citations, represented through a co-citation network. The distribution of journals, their Impact Factor (IF, 2022), and categories published by Journal Citation Reports (JCR, 2022) were also analyzed, along with the production and distribution of top journals. Furthermore, the study explored the distribution of resources and subjects in the publications. It calculated the H-index for authors or institutions and conducted an analysis of keyword frequencies and their co-occurrence network. Finally, keywords were subjected to cluster analysis to identify related themes. This comprehensive analysis aimed to provide valuable insights into the trends and characteristics of research in the field of AAV gene therapy.

In this study, we employed various tools and software for quantitative examination and analysis in scientometrics. The online analysis platform (https://bibliometric.com/), R language (version 3.6.3), bibliometrix (version 3.1.4), Citespace (version 6.3R3), VOSviewer (version 1.6.18), and Microsoft Excel 365 were utilized to analyze different aspects of the research data. Microsoft Excel 365 was used for quantitative analysis of articles, journals, authors, countries, and institutions. VOSviewer 1.6.18, a freely available tool, was used to create and visualize bibliometrics networks and maps based on data such as journal relationships, author collaborations, country affiliations, and citation patterns. This facilitated the identification of research hotspots in the field by extracting keywords from bibliometric co-occurrence analysis. Citespace (version 6.3. R3) was utilized to produce visual maps on journals, authors, countries, institutions, keywords, and document citations in each divided time zone. In these graphs, each node type and size represented the weight of each parameter, such as the occurrence frequency of keywords and the quantity of published articles for other parameters. The lines linking nodes represented the strength of relationships between parameters. Clustering analysis and time zone views were also performed to identify local mutation words, research hotspots, and trends. Three clustering algorithms (latent semantic indexing - LSI, log-likelihood ratio - LLR, and mutual information - MI) were used. In this study, the variables were presented as numbers and percentages, and no comparisons were made, hence no P-values were reported. The focus was on providing a comprehensive overview of the data without statistical testing.

## 3 Results

### 3.1 The trend of publication outputs

**Figure 2:**
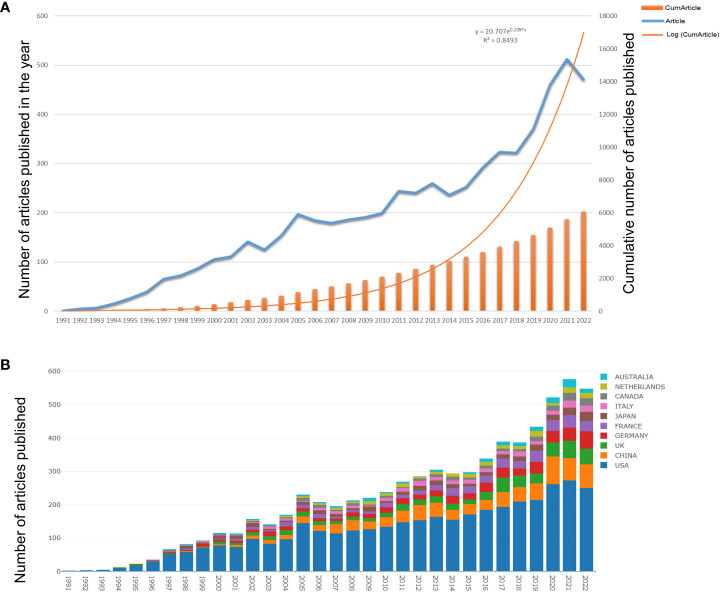
Global trends in publications on genetic therapy and AAV. **(A)** Annual publication growth trend. Equation: y = y=20.707e^0.2097x^, R2 = 0.8493 can predict the annual documents. **(B)** Dynamics of publications in the top 10 countries from 1991 to 2022. The quantity of publications was on the rise.

Based on the inclusion criteria of publication time, type, and language, a total of 6,069 articles were available for further analysis. The temporal distribution of publications related to AAV and genetic therapy is depicted in **Figure 2A**. Over the three decades studied, the total number of annual papers has consistently increased, displaying an exponential growth pattern. The exponential curve equation, y=20.707e^0.2097x^, with a high coefficient of determination (R^2^ = 0.8493), indicates a strong upward trend in future annual publications. Notably, the field has seen an exponential surge in research output since 2010, with the highest number of studies published in 2021. These findings collectively demonstrate a growing focus on AAV and genetic therapy within the broader domain of gene therapy.

### 3.2 Distribution of countries/regions

According to the inclusion criteria, a total of 25,319 authors from 3,809 institutions published their work in 1,053 academic journals across 75 countries and regions. **Table 1** presents the top ten countries contributing to these publications. The United States leads with the highest number of articles, contributing 3,868 articles (63.73%), followed by China with 707 articles (11.65%) and the United Kingdom with 478 articles (7.88%). The publications from the United States also garnered the highest number of citations, totaling 205,547, indicating their significant international influence. Scholars from the United States achieved the highest H-index, further reinforcing their research’s substantial impact. **Figure 2B** illustrates the total number of articles published by the top 10 countries from 1991 to 2022. In recent years, China, England, Germany, and France have shown a significant increase in the number of publications, reflecting a notable percentage change in their research output.

**Table 1:** The top 10 countries that contributed publications on genetic therapy and AAV vectors.

Rank	Countries	Article count	Percentage (n/6069)	Total citations	Average citation per article	H-index
1	USA	3,868	63.73%	205,547	53.14	187
2	China	707	11.65%	13,085	18.51	52
3	England	478	7.88%	20,628	43.15	65
4	Germany	473	7.79%	18,247	38.58	73
5	France	383	6.31%	16,183	42.25	64
6	Japan	289	4.76%	7,991	27.65	48
7	Italy	230	3.79%	12,124	52.71	58
8	Canada	192	3.16%	9,625	50.13	49
9	Netherlands	181	2.98%	8,728	48.22	49
10	Australia	167	2.75%	5,412	32.41	38

**Figure 3:**
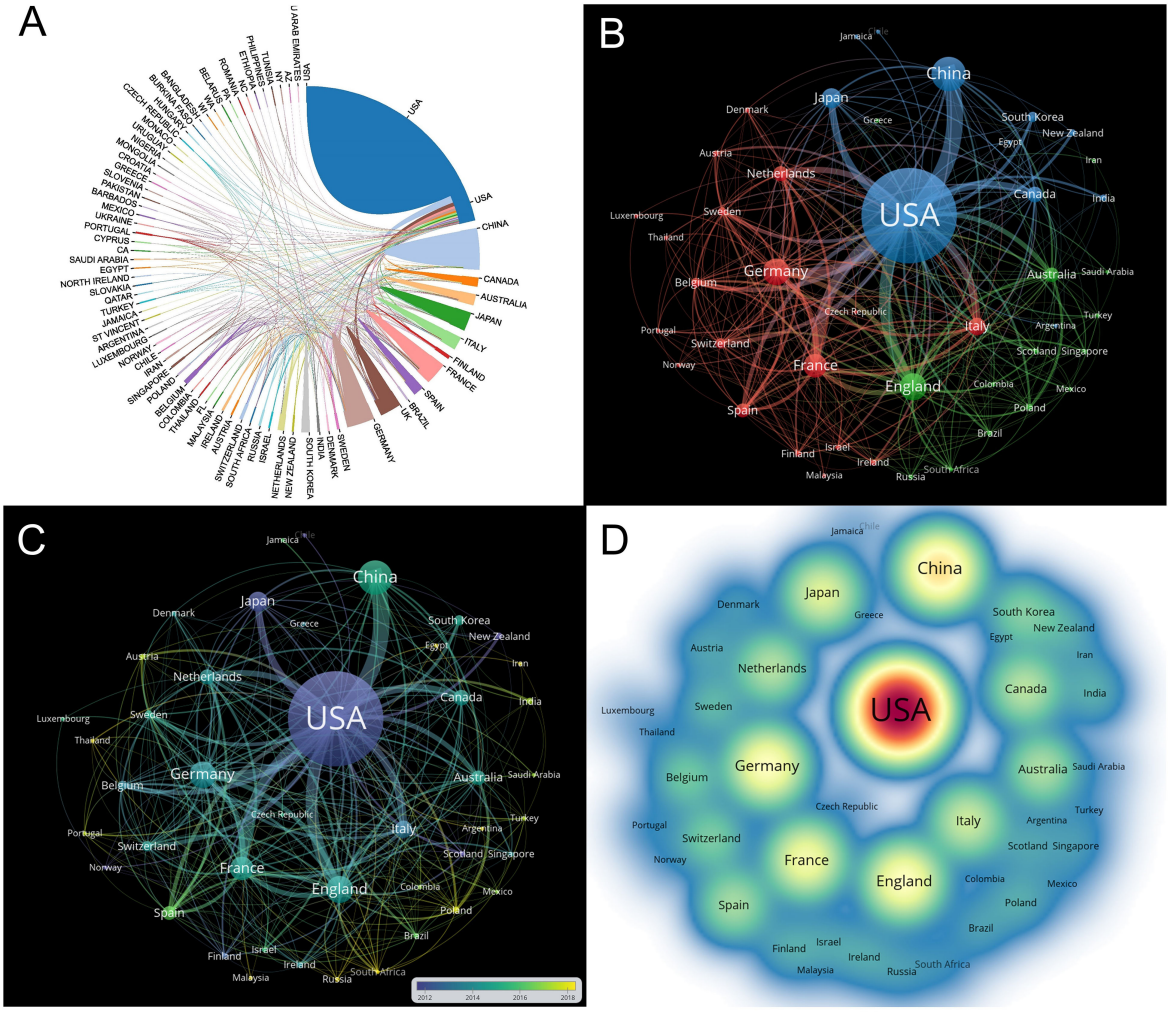
National publications overview. **(A)** The collaboration of countries. Each country is depicted by a block of color, with larger blocks representing a greater number of publications. The line between the blocks signifies international cooperation. The thicker the line, the stronger the collaboration. **(B)** Citation relationships between countries/regions. Minimum number of documents per country ≥5. The size of the nodes represented the number of publications, while the thickness of the linkages represented the citation strength. **(C)** The map of citation relationships between countries over time. **(D)** Density visualization of countries/regions. Word size, circle size, and red opacity are correlated with higher number of documents.

**Figure 3A** shows the collaborative relationship networks between countries, with the United States having the most positive influence, followed by China, Germany, the UK, and France. The United States and China exhibit the closest collaborative ties with numerous other countries. Using VOSviewer, **Figure 3B** illustrates the citation relationships between 45 countries, with a minimum document threshold of 5 for screening. The visualization in **Figure 3C** depicts the citation relationships of each country over time. Publications from the United States and Japan were prominent around 2012, while those from China, India, and South Korea were concentrated around 2016. Furthermore, the density map in **Figure 3D** reflects the number of articles from each country, with word, circle, and red opacity size correlated with the number of documents. The major contributors to the field of AAV and genetic therapy are the United States, China, Germany, the UK, and France. In conclusion, these visualizations provide insights into the collaborative networks and significant contributions of various countries in the research on adeno-associated viruses and genetic therapy.

### 3.3 Distribution of institutions

**Table 2** presents the top 10 institutions in terms of publication ranking, showcasing their dominant positions in the field of AAV and genetic therapy. Notably, 90% of these top institutions are based in the United States, with a solitary institution from France representing the remaining 10%. The State University system of Florida from the US holds the highest article count, contributing 636 articles (10.48%), followed closely by the University of Florida with 605 articles (9.97%), and the University of Pennsylvania with 474 articles (7.81%). The University of Pennsylvania, also from the United States, stands out with the highest H-index of 108, the highest total citations of 43,449, and the highest average citation per article at 91.66. These impressive metrics demonstrate the institution’s significant influence and recognition within the field, as evidenced by the impact of its publications.

**Table 2:** The top 10 institutions that contributed publications on genetic therapy and AAV vectors.

Rank	Institutions	Countries	Article count	Percentage (n/6069)	Total citations	Average citation per article	H-index
1	State University System of Florida	USA	636	10.48	37,904	59.6	97
2	University of Florida	USA	605	9.97	36,758	60.76	97
3	University of Pennsylvania	USA	474	7.81	43,449	91.66	108
4	Pennsylvania Medicine	USA	325	5.35	29,192	89.92	87
5	University of North Carolina	USA	311	5.12	23,317	74.97	77
6	University of North Carolina Chapel hill	USA	309	5.09	23,225	75.26	77
7	University of California System	USA	307	5.06	19,172	62.45	77
8	Institut national DE LA Sante ET DE LA Recherche Medicale Inserm	France	275	4.53	10,673	38.81	59
9	Harvard University	USA	274	4.51	15,367	56.08	67
10	National Institutes of Health NIH USA	USA	251	4.14	16,955	67.55	66

**Figure 4:**
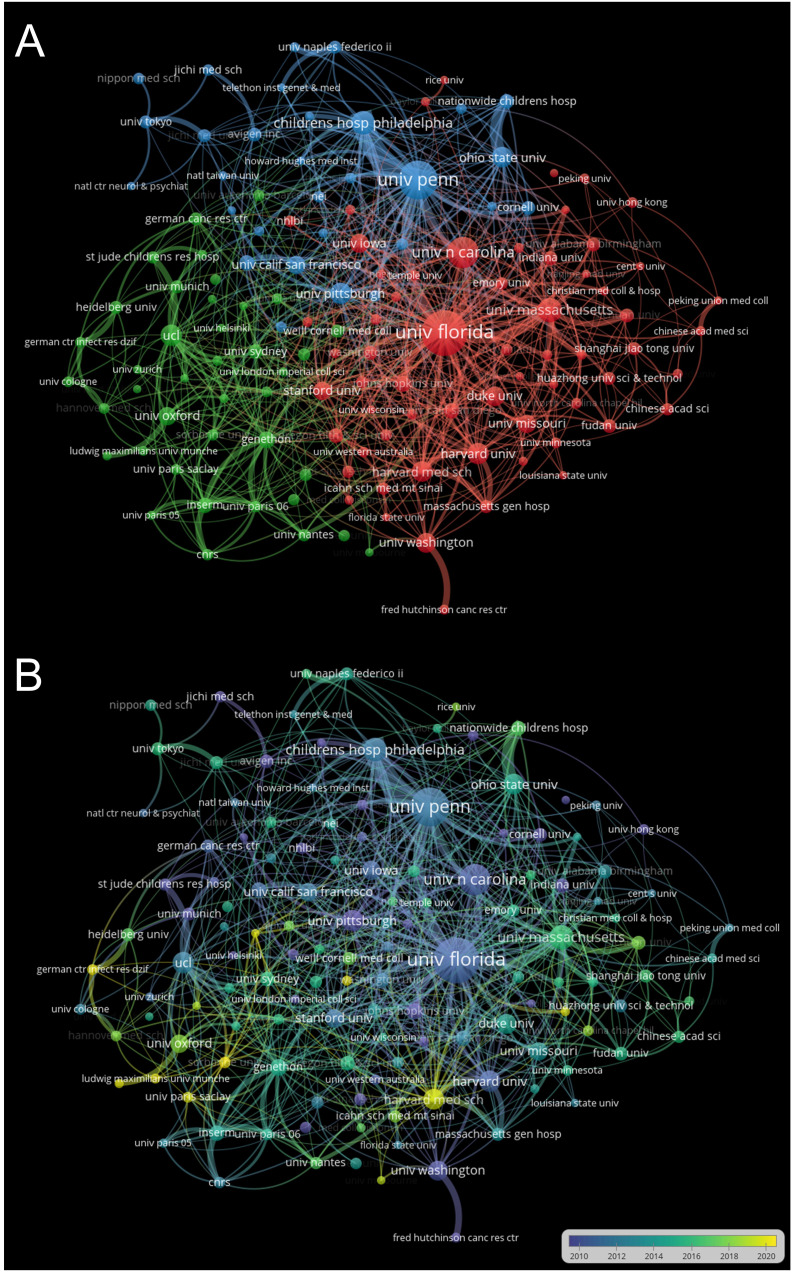
Cooperation networks between institutions. Minimum number of documents per organization ≥20. **(A)** Cooperation relationship chart of various institutions. The size of the nodes indicated the number of publications, while the thickness of the lines showed the strength of the collaboration. **(B)** Cooperation time chart of various institutions. Recent institutional collaborations are highlighted in yellow.

The study involved 133 organizations as nodes, screened with a minimum of 20 documents as the threshold. The resulting graphical representation in **Figure 4A** depicted the cooperative relationships between institutions, forming three clusters comprising 133 nodes. The thickness of the lines indicated the strength of collaboration between two institutions, with the University of Florida, University of Pennsylvania, and University of North Carolina displaying a strong collaborative relationship. The node size reflected the number of documents, with the University of Florida having the largest node size due to its high document count. **Figure 4B** illustrated the institution cooperative relationships over time. Yellow nodes highlighted present institutional collaborations, exemplified by the active partnerships involving the Harvard Medical School. On the other hand, blue nodes denoted past institutional collaborations, such as the University of Pittsburgh’s prior collaborative efforts. These visualizations offered valuable insights into the collaborative networks among institutions in the field of AAV and genetic therapy research. They emphasized the strength and dynamic nature of collaborative endeavors in advancing scientific knowledge in this domain.

### 3.4 Authors and co-cited authors

**Figure 5:**
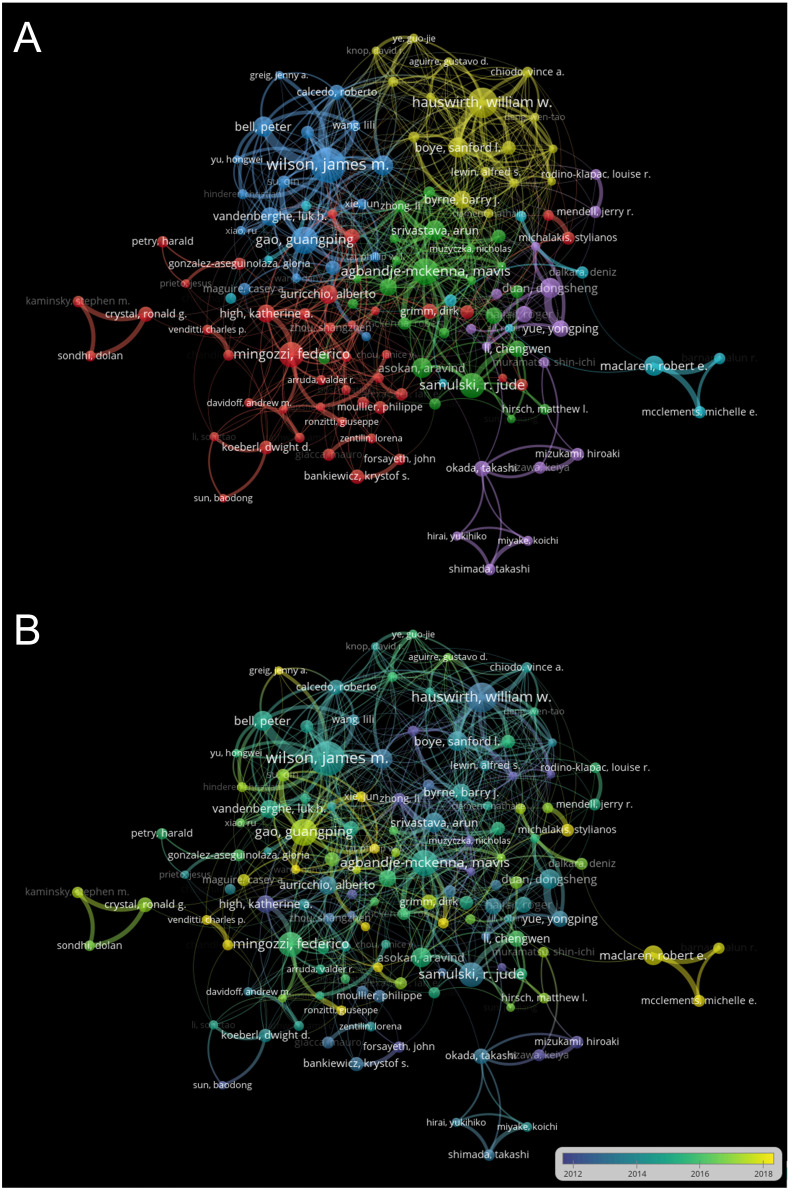
Visualization of author analysis. **(A)** Author collaboration network analysis. Minimum number of documents per author ≥15. The size of the nodes indicated the number of articles published by the author, while the thickness of the lines showed the strength of the collaboration. **(B)** Cooperation time chart of of author collaboration. The nodes indicated with purple or blue color represent earlier-appearing author collaboration, while the author collaboration marked with yellow color show current research concerns.

**Table 3** lists the top 10 contributing authors and the top 10 co-cited authors. Wilson JM from the University of Pennsylvania, the United States, ranks first with 166 articles (2.73%), followed by Samulski RJ from the University of North Carolina, the United States (151, 2.48%), and Hauswirth WW from the University of Florida, the United States (123, 2.03%). Co-cited authors refer to two or more authors cited together in one or more papers. Samulski RJ stands out with the highest total citations (8,067), indicating significant recognition. The collaborative network between authors, screened with a minimum of 15 documents per author, is depicted in **Figure 5A**, consisting of 6 clusters and 163 nodes. In **Figure 5B**, the current authors’ collaborations around 2018 are highlighted, with Gao GP having the most collaborations in the current research study, denoted by yellow nodes.

**Table 3:** The top 10 authors and co-cited authors that contributed publications on genetic therapy and AAV vectors.

Rank	Author	Article count	Total citations	Average citation per article	H-index	Countries	Institutions	Co-cited author	Citations	Countries	Institutions
1	Wilson JM	166	16,345	98.46	67	USA	University of Pennsylvania	Samulski RJ	8,067	USA	University of North Carolina
2	Samulski RJ	151	17,594	116.52	46	USA	University of North Carolina	Wilson JM	7,183	USA	University of Pennsylvania
3	Hauswirth WW	123	9,142	73.14	50	USA	University of Florida	Mingozzi F	5,526	France	Genethon
4	Flotte TR	112	9,724	86.82	50	USA	University of Massachusetts	High KA	5,227	USA	Spark Therapeutics
5	Gao GP	108	9,535	88.29	46	USA	University of Massachusetts	Gao GP	4,461	USA	University of Massachusetts
6	Agbandje MM	84	5,166	61.5	40	USA	University of Florida	Flotte TR	3,653	USA	University of Massachusetts
7	Mingozzi F	81	9,408	116.15	44	France	Genethon	Hauswirth WW	3,463	USA	University of Florida
8	Duan Ds	64	5,205	76.54	39	USA	University of Missouri	Xiao X	3,280	USA	University of North Carolina
9	Byrne B	62	6,510	105	34	USA	University of Florida	Muzyczka N	3,173	USA	University of Florida
10	Maclaren RE	56	1,747	31.2	22	England	University Of Oxford	Calcedo R	3,154	USA	University of Pennsylvania

### 3.5 Journals and co-cited journals

**Figure 6:**
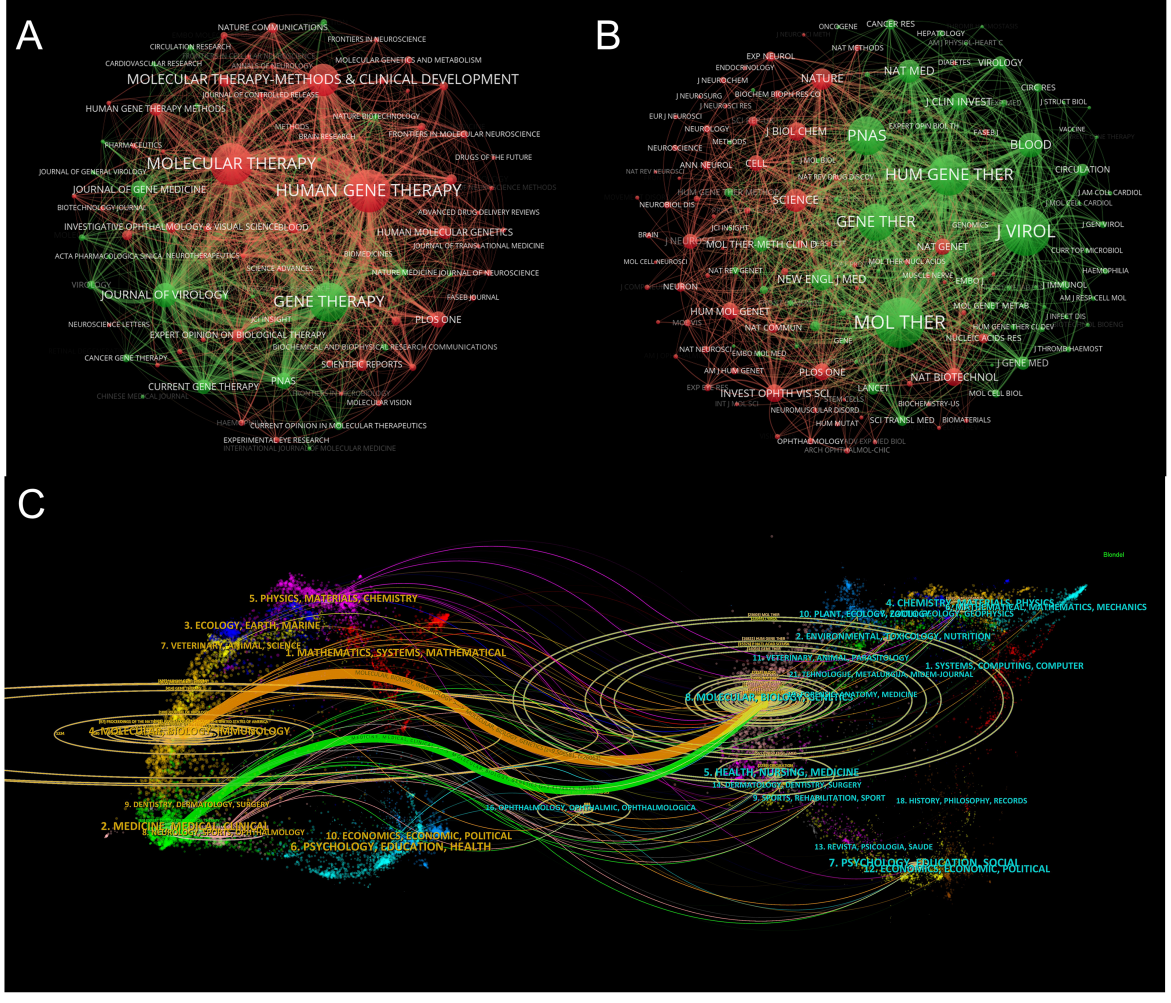
Analysis of journals involved in Genetic therapy and AAV. **(A)** The visualization of journals. Minimum number of documents per journal ≥10. As the largest node in the graph, Experimental Gerontology has the most publications. **(B)** The visualization of co-cited journals. Minimum number of citations per journal ≥300. The size of the node is proportional to co-citation frequency, the Journal of Immunology has the most co-citations. **(C)** The dual-map overlay of journals related to research of Genetic therapy and AAV. The left side contained the citing journal, the right side contained the cited journal, and the path of the line reflected the citation connection.

**Table 4** presents the top 10 contributing journals and the top 10 co-cited journals in the field of adeno-associated viruses and genetic therapy. Leading the list, Human Gene Therapy published 485 articles (7.99%), followed by Molecular Therapy (472, 7.78%) and Gene Therapy (414, 6.82%). **Figure 6A** provides a visible analysis map of journals, with node size corresponding to the number of articles published and link line thickness indicating the strength of collaboration between two journals. Regarding co-cited journals (**Table 4**), Molecular Therapy ranks first with a total of 23,603 co-citations, followed by Journal of Virology with 22,644 co-citations and Human Gene Therapy with 16,821 co-citations. **Figure 6B** illustrates the visualization network of 145 co-cited journals with more than 300 citations, with node size reflecting the frequency of co-citations. Additionally, **Figure 6C** showcases the dual-map overlay of articles citing adeno-associated viruses and genetic therapy research. The color link routes indicate relationships between the left field of the citing journal and the right field of the cited journal. Journals in the field of Molecular/Biology/Genetics were frequently cited in molecular/biology/immunology journals, shown by orange pathways. Journals primarily focused on Molecular/Biology/Genetics research were cited in Medicine/Medical/Clinical, indicated by green pathways. These analyses offer valuable insights into the prominence and influence of journals in the domain of AAV and genetic therapy research and highlight the interdisciplinary nature of the field.

**Table 4:** The top 10 journals and co-cited journals that published papers on genetic therapy and AAV vectors.

Rank	Journals	Counts	Total citations	Average citation per article	IF and JCR division	Co-cited Journals	Total co-citations	IF and JCR division
1	Human Gene Therapy	485	19,598	40.41	4.2, Q1/ Q2	Molecular Therapy	23,603	12.4, Q1
2	Molecular Therapy	472	32,425	68.7	12.4, Q1	Journal of Virology	22,644	5.4, Q2
3	Gene Therapy	414	20,498	49.51	5.1, Q2/Q3	Human Gene Therapy	16,821	4.2, Q1/ Q2
4	Molecular Therapy Methods Clinical Development	299	4,958	16.58	4.7, Q2	Proceedings of the National Academy of Sciences of the United States of America	15,376	11.1, Q1
5	Journal of Virology	188	18,757	99.77	5.4, Q2	Gene Therapy	14,054	5.1, Q2/Q3
6	Journal of Gene Medicine	102	3,108	30.47	3.5, Q2/Q3	Blood	7,324	20.3, Q1
7	Plos One	97	2,624	27.05	3.7, Q2	Science	6,530	56.9, Q1
8	Proceedings of the National Academy of Sciences of the United States of America	97	13,320	137.32	11.1, Q1	Nature Medicine	6,495	82.9, Q1
9	Current Gene Therapy	80	2,617	32.71	3.6, Q2	Nature	5,686	64.8, Q1
10	Human Molecular Genetics	69	3,023	43.81	3.5, Q1/Q2	New England Journal of Medicine	5,617	158.5, Q1

### 3.6 Co-cited references and references burst

**Figure 7:**
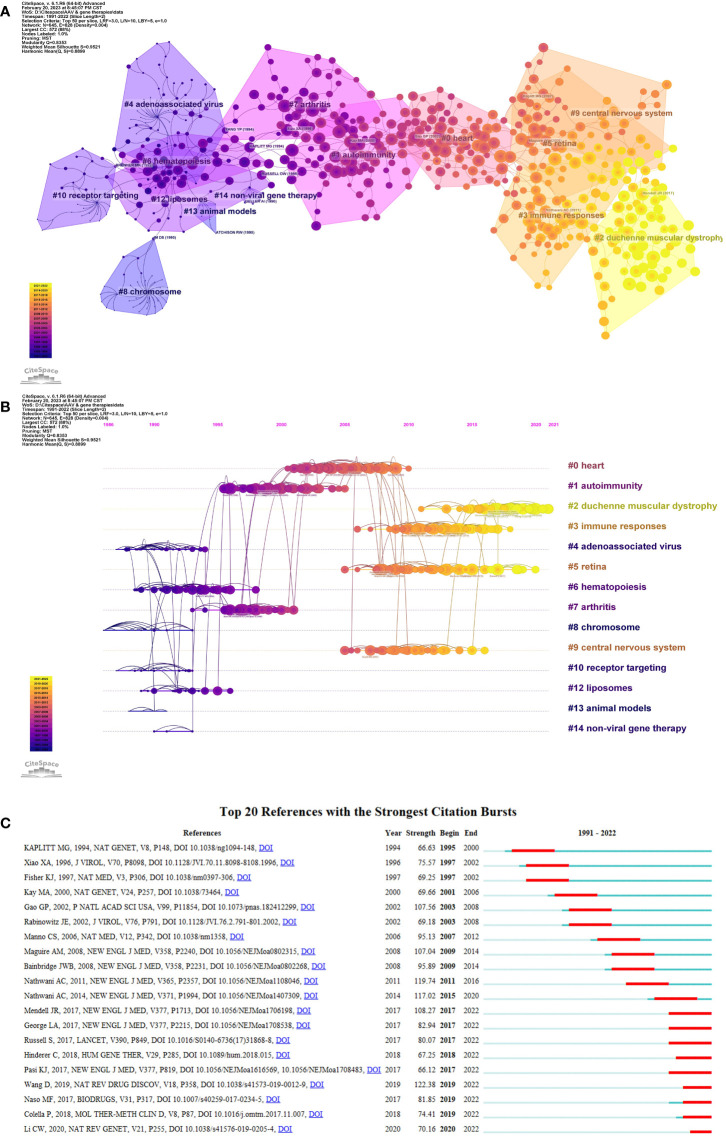
Co-citation analysis of references. **(A)** The co-citation clusters of co-cited references. The figure describes 14 color blocks, each indicating a cluster of articles on the same subject. By default, clusters with fewer than ten articles were filtered out and not displayed. **(B)** The timeline view of references related to genetic therapy and AAV research. fourteen labeled clusters are colored on the right. The nodes on the line indicated the cited references. The labels indicated with purple represent earlier appearing clusters, while the clusters marked with yellow color show current research concerns. **(C)** The top 20 references with the strongest citation bursts on research of Genetic therapy and AAV.

The frequency of citations often reflects the importance and relevance of a document’s topic. **Supplementary Table 1** presents the top 20 most cited literature, with a minimum of 265 local citations for each reference. In **Figure 7A**; **Supplementary Table 2**, these references are grouped into 14 clusters. **Figure 7A** describes different color blocks of clusters, and each clusters represents a distinct research subject. Clusters with fewer than ten articles were filtered out and not displayed, for instance, cluster #11. **Figure 7B** displays a timeline where subject clusters are concentrated in specific time ranges. Purple labels on the line indicate earlier occurrences of subjects such as #8 chromosome and #10 receptor targeting. In contrast, yellow cluster nodes represent current research subjects like #2 Duchenne muscular dystrophy and #5 retina. The reference with the highest number of global citations (1599) experienced a notable citation burst from 2009 to 2014, as depicted in **Figure 7C**; **Supplementary Table 1**. This reference, published in the New England Journal of Medicine in 2008 by Maguire AM et al., evidently attracted substantial attention during that period (Maguire et al., 2008).

### 3.7 Analysis of keywords and hotspots

Keywords serve as essential indicators of an article’s core topic, reflecting the trends and hotspots in a specific academic field. We conducted keyword cluster analysis and obtained eleven clusters (**Supplementary Table 3**), reflecting changes of research trends over time. “vectors”, “adenoassociated virus” and “retrovirus vectors” were large clusters with multiple articles, the keywords “safety” had the most recent outburst citations.

**Figure 8:**
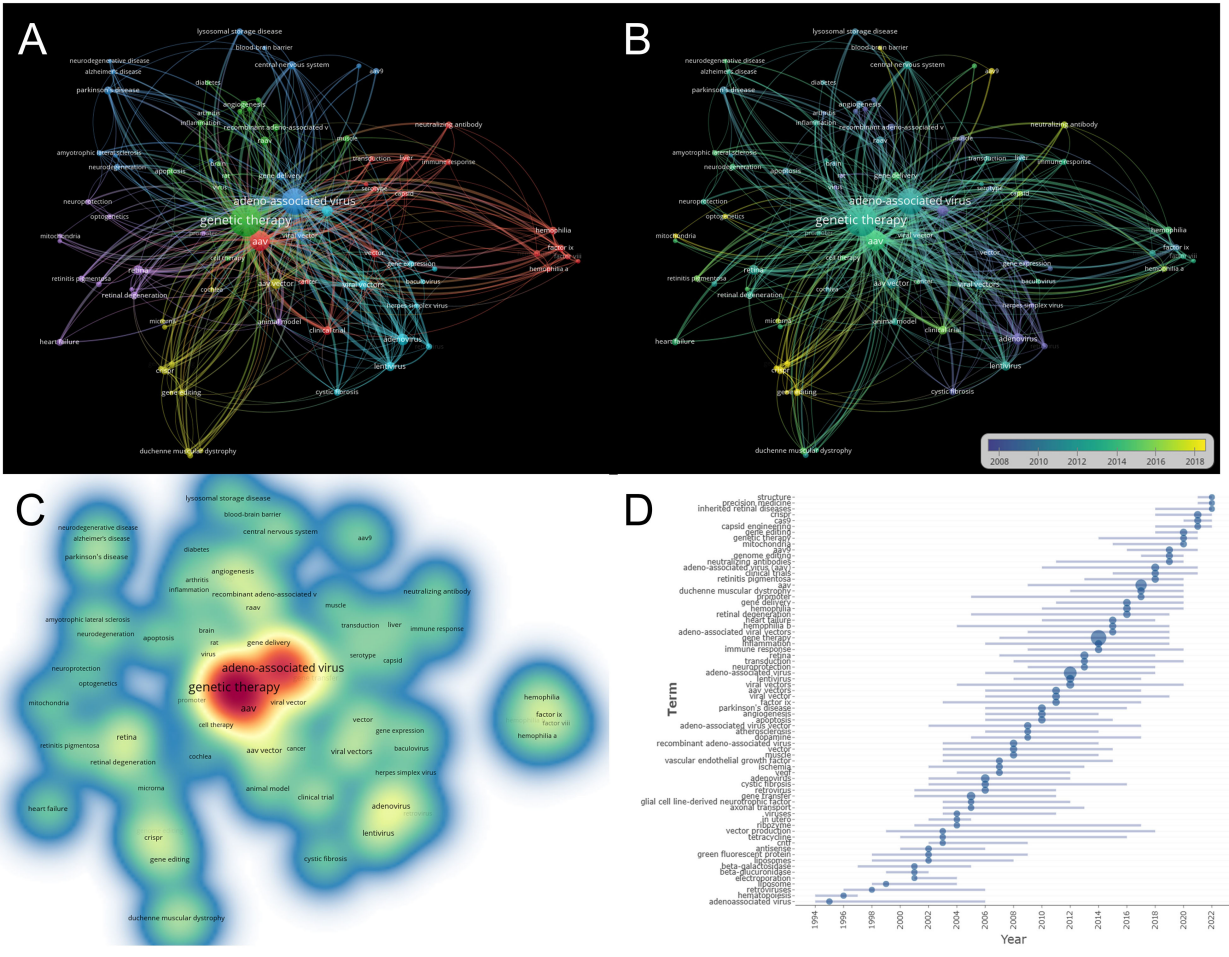
Visualization of keyword analysis. **(A)** Items with occurrences more than 20 times were shown in the network map of co-occurrence keywords links. **(B)** Overlay of co-occurrence keywords. The nodes of keywords with purple or blue color indicated keywords that appeared earlier, while those with yellow color indicated current research concerns. **(C)** The density visualization of keywords. Word size, circle size, and red opacity are correlated with higher frequency of keywords occurrence. **(D)**The timeline view of keywords related to genetic therapy and AAV research.

In our study, we set a minimum occurrence threshold of 20 for keywords and identified 83 relevant items meeting the criteria. The network map of co-occurrence keyword links (**Figure 8A**) presents 6 clusters based on the strength of keyword co-occurrences. In **Figure 8A**, the green cluster prominently features keywords related to genetic therapy, while the blue cluster contains keywords associated with adeno-associated viruses. Notably, genetic therapy and adeno-associated viruses are located in separate clusters in terms of co-occurrence. **Figure 8B** further highlights the keywords with citation bursts between 1994 and 2022. In recent years, there has been a growing emphasis on AAV-related research involving CRISPR gene editing technology and clinical applications for various diseases, including ophthalmological, hematological, muscular, cardiovascular, and central nervous system disorders. Moreover, the density visualization of keywords (**Figure 8C**) emphasizes the most frequent occurrences of “genetic therapy” and “adeno-associated viruses,” underlining their significance in this research field. These visualizations provide valuable insights into the prominent keywords and emerging research trends in adeno-associated viruses and genetic therapy. **Figure 8D** presents AAV-related studies have garnered significant attention in the fields of gene structure, precision medicine, inherited retinal disease, and CRISPR/Cas9-mediated gene editing.

### 3.8 Analysis of AAV gene therapy latest researches in 2023

We have continued to supplement the relevant literature on AAV gene therapy, with a total of 428 publications up to November 12, 2023. Regarding the contributions of the authors, Gray SJ from the University of Texas Southwestern Medical Center in the United States leads the list with 8 articles, followed by Chamberlain JS with 6 articles from the University of Washington in the United States, Gao GP with 5 articles from the University of Massachusetts in the United States, and Duan DS with 5 articles from the University of Missouri in the United States. McKenna R, also from the United States, has 5 articles affiliated with the University of Florida. Regarding the contributing journals, topping the list was Human Gene Therapy published 37 articles (8.64%), followed by Molecular Therapy Methods and Clinical Development with 25 articles (5.84%) and International Journal of Molecular Sciences (25, 5.84%). **Supplementary Table 4** presents the top 10 most cited literature, with a maximum of 21 citations in global field. The article “Gene Therapy with Etranacogene Dezaparvovec for Hemophilia B” by Pipe SW has garnered widespread citations globally, locating the top position in citation counts.

## 4 Discussion

### 4.1 General information

Bibliometric analysis is widely used to provide a comprehensive overview of academic fields and identify trends and hotspots. In this study, we analyzed a total of 6,069 literature publications from 1991 to 2022, published in 1,053 academic journals, authored by 25,319 individuals from 3,809 institutions across 75 countries/regions, based on the WoSCC database. The increasing number of publications on Adeno-associated virus and gene therapy research indicates AAV as a research hotspot in gene therapy.

Regarding country/region distribution, the United States demonstrated a dominant position with the highest number of publications and citations, as well as the highest H-index among scholars. Consequently, the United States contributed significantly to this field. Notably, the University of Florida emerged as the institution publishing the largest number of articles on AAV and gene therapy. Their recent studies focused on an efficient approach using mitochondrially targeted Adeno-Associated-Virus (MTS-AAV) for Leber’s hereditary optic neuropathy (Liu et al., 2022). However, it is observed that most of the top 10 contributing institutions are from the United States, suggesting a limitation in international academic development in this field. To promote research in Adeno-associated virus and gene therapy, academic institutions should strengthen collaboration and communication among different regions.

Turning to the top 10 contributing authors and co-cited authors (**Table 3**), Wilson, James M exhibited significant influence with the most published literature and also ranked in the top 3 for total citations and co-citations, underscoring his impact in AAV and gene therapy. In one of their recent publications, Wilson’s team explored the role of Neonatal Fc receptor (FcRn) in AAV-based gene therapies, highlighting its impact on neutralizing antibodies (NAbs). They assessed the efficacy of an FcRn-inhibiting monoclonal antibody (M281) to improve AAV-based therapies in patients with pre-existing immunity (Horiuchi et al., 2023). On the other hand, Samulski, R. Jude had the most total citations and co-citations, and his recent review highlighted advancements in AAV vectors for gene therapy, providing valuable insights into the current state of AAV-based gene therapies (Pupo et al., 2022).

Among the top 20 locally cited articles, the majority were articles, indicating that AAV is a promising gene therapy tool for achieving efficient and long-term *in vivo* gene transfer in hereditary diseases (Xiao et al., 1998; Gao et al., 2002; Maguire et al., 2008; Nathwani et al., 2011). Among these highly cited articles, two stood out with the most references. The first article, “Novel adeno-associated viruses from rhesus monkeys as vectors for human gene therapy” by Gao GP, published in 2002, presented the use of adeno-associated viruses from rhesus monkeys as vectors for human gene therapy (Gao et al., 2002). The study utilized multiple mouse models to assess the efficacy of AAV-mediated gene transfer in skeletal muscle and liver, effectively demonstrating the relative performance of the vectors. The second highly cited article, “Safety and efficacy of gene transfer for Leber’s congenital amaurosis” by Maguire AM et al. investigated the use of adeno-associated virus (AAV) carrying RPE65 complementary DNA (cDNA) for gene therapy in patients with Leber’s congenital amaurosis (Maguire et al., 2008). The study observed a slight improvement in retinal function in patients treated with the AAV-based gene therapy, highlighting its potential for therapeutic applications. Additionally, through cluster analysis and timeline view analysis of the references, it was observed that recent references have focused on specific areas, including duchenne muscular dystrophy, retina-related studies, immune responses, and the central nervous system (**Figure 7B**). These findings reflect the ongoing research interests and current research trends in the field of adeno-associated viruses and genetic therapy.

### 4.2 The hotspots and trends

It can be seen from **Figure 8D** (keywords with reference bursts) and **Figures 8A, B** (co-occurrence analysis of keywords) that AAV gene therapy research has involved gradually in clinical inherited disease treatment applications, which can reveal the research hotspot and development trend in this field. we can conclude that gene editing and disease were recently study hotspots.

#### 4.2.1 Structure and feature of AAV

AAV is a single-stranded DNA virus with replication defects, requiring the assistance of a helper virus like adenovirus or herpes simplex virus to complete the transfection process. However, recent advancements have led to improved AAV vectors that no longer require the assistance of adenovirus, making them highly desirable for gene therapy due to their high transfection efficiency and safety profile. The structure of AAV is characterized by its small size, approximately 20~26 nm in diameter, and a genome size of about 4,700 bp (Daya and Berns, 2008; Samulski and Muzyczka, 2014). The AAV genome contains the rep and cap genes flanked by two inverted terminal repeats (ITRs) (Wu et al., 2006; Hastie and Samulski, 2015). The rep genes are responsible for AAV genome replication and virion assembly, while the cap gene encodes three capsid proteins (VP1, VP2, and VP3) that form the AAV virion (Li and Samulski, 2020). AAV virions consist of 60 VP subunits at a specific ratio, and each subunit has nine variable regions on its surface, which can be genetically modified to alter the transduction efficiency and immune response (Xie et al., 2002; Govindasamy et al., 2006; Büning et al., 2015; Li and Samulski, 2020). Importantly, the 145bp AAV ITRs are essential for inducing transgene expression, ensuring the stability of the vector and long-lasting cell transduction. As a result, the design of recombinant AAV (rAAV) vectors involves removing most of the viral genome, leaving behind only the necessary elements, such as promoters, transgenes, and poly A tails, for gene therapy applications (Wu et al., 2008). Currently, there are 13 serotypes of AAV that have been isolated and can be used as gene delivery vectors. Each serotype offers unique properties and advantages, making AAV a versatile tool in gene therapy with various modifiable elements (Kotterman and Schaffer, 2014; Li and Samulski, 2020). Overall, the high biosafety, wide host range, long expression time, and low immunogenicity of AAV make it a valuable and promising vector for gene therapy applications.

#### 4.2.2 AAV and CRISPR associate proteins

Clustered regularly interspaced short-palindromic repeat (CRISPR) associated (Cas) proteins, which are guided by a short RNA sequences that can be identified by Watson-Crick base pairing, are widely used in gene editing research (Brouns et al., 2008; Jinek et al., 2012). Cas nickase (nCas)-based methodologies, including base editing and prime editing, have introduced the potential to create minor modifications without involving DNA double-strand breaks (DSB) and homology-directed repair (HDR) engagement. This ensures a more uniform genetic outcome at the designated target sites, and Fiumara et al. found that CBE, ABE, and PE reduce but do not eliminate the occurrence of DNA DSBs at their genomic target sites, exposing cells to the potential genotoxic effects of deletions and translocations in contrast to conventional Cas nuclease-based editing. (Anzalone et al., 2020; Fiumara et al., 2023). The rapid development of CRISPR technology has been applied to human genetically related diseases, and it holds great prospect for treating many genetic diseases. CRISPR-based technology is capable of handling a wide range of gene editing operations, and approaches of delivering it by AAV vector remains a hot topic in therapeutic development (Wang et al., 2019), such as limitations in the packaging size of AAV vectors and non-targeted editing results occur. Ran et al., 2015 and Kim et al., 2017 identified smaller Cas9 lineal homologues, packaged them and single guide RNA (sgRNA) expression box into a AAV vector *in vivo* (Ran et al., 2015; Kim et al., 2017). Tornabene and Trapani, 2020 summarized the most studied approach is the use of multiple single vectors, into which the large, segmented transgenes were loaded (Tornabene and Trapani, 2020). The AAV vector delivers the split intron of reconstruction, and the targeted protein expression is reprogrammed (Chew et al., 2016). Realizing the benefits of gene therapy through synergies between CRISPR therapy and AAV-mediated delivery (Wang et al., 2020). In conclusion, CRISPR technology’s synergy with AAV-mediated delivery, despite existing challenges, presents promising advancements in the treatment of genetic diseases. Currently, the CRISPR-Cas9 gene editing technology has achieved significant milestones, making the treatment of numerous genetic diseases possible. However, according to some researches, there are still technical safety concerns, such as off-target activity, immune response and the persist of chromosomal translocation (Allen et al., 2018; Wagner et al., 2019; Samuelson et al., 2021; Tsuchida et al., 2023). For the application of AAV and other delivery vectors combined with CRISPR, there exists a noticeable concern where pre-existing immunity diminishes the *in vivo* delivery efficiency (Vandamme et al., 2017; Patsali et al., 2019).

#### 4.2.3 AAV and hereditary diseases

Hereditary diseases are due to the deletion of a gene, chromosome mutation and other genetic material changes that lead to abnormal genetic coding of functional proteins. Therefore, The gene therapy is a approach of the target gene integrated into the patient genome to restore normal gene expression. It has become a hot topic rapidly evolving in the treatment of genetically related diseases. Adeno-associated virus is one of the most widely applied viral vectors in gene therapy.

##### 4.2.3.1 The application of AAV in ophthalmologic diseases

In past studies, the genetic factors of certain retinal diseases have been identified, which provides a basis for the application of gene therapy in genetically related retinal diseases (Rodrigues et al., 2018). The causative genes have been identified in inherited retinal diseases (IRDs) such as retinitis pigmentosa (RP) and cone and rod dystrophies, LCA or achromatopsia, Stargate disease and Best diseases (Farrar et al., 2017).These drugs are injected subretinal in patients with RPE65-Leber congenital amaurosis using AAV2 vector carrying a functional copy of the retinal pigment epithelium (RPE) specific 65-kDa protein gene (RPE65) in 2008, and finally, it is demonstrated that visual function improved in these trials (Bainbridge et al., 2008; Hauswirth et al., 2008; Maguire et al., 2008).At the same time, many pathogenic or susceptibility genes related to other retinal diseases like AMD and glaucoma were also found. The role of VEGF has been confirmed in the occurrence and development of neovascular AMD. However anti-VEGF drug therapy requires monthly injections to maintain efficacy. Gene therapy with AAV combined with anti-VEGF drug injection has the possibility of prolonging the action time of the drug (Constable et al., 2017; Heier et al., 2017). The AAV vector has demonstrated promise in ophthalmologic applications, particularly in genetic retinal diseases, by enhancing treatment efficacy and duration.

##### 4.2.3.2 The application of AAV in hematological diseases

Hemophilia is an X-linked congenital hemorrhagic disorder caused by F8 or F9 single gene mutations that cause secretion insufficiency of coagulation factor VIII (FVIII; Hemophilia A) Or clotting factor IX (FIX; Hemophilia B) (Berntorp et al., 2021). To date, most studies based on AAV gene therapy have been conducted in patients with hemophilia B, because the length of FVIII gene is too large to fit into AAV vector, which limits the application of the vector in hemophilia A gene therapy (Leebeek and Miesbach, 2021). In 2017, the FVIII gene trial of intravenous AAV5 (valxaparvovec) was reported in patients with hemophilia A. Long-term data after 3 years of follow-up showed a significant reduction in FVIII expression compared to the FVIII level at 1 year (Pasi et al., 2020). In 2006, researchers conducted the first clinical study of AAV2 vector liver gene therapy in patients with severe hemophilia B, and FIX expression was sustained for 2 months through hepatic artery infusion vector (Manno et al., 2006). In order to achieve higher levels of FIX activity, an AAV FIX Padua (FIX R338L) construct, fidanacogene elaparvovec (SPK9001; Spark), was developed that contains FIX variants that are five to eight times more active (George et al., 2017). It is important to note that there are problems with variability in expression levels, the occurrence of immune responses, and the need for immunosuppressive therapy due to the occurrence of abnormal liver function (Leebeek and Miesbach, 2021). Intravenous AAV liver targeted gene therapy in patients with hemophilia A and B can increase levels of FVIII and FIX, even reaching the normal range, achieving significant therapeutic efficacy. In conclusion, AAV-based gene therapy offers significant therapeutic potential for hemophilia, despite existing challenges like immune responses and variable expression levels.

##### 4.2.3.3 The application of AAV in muscular disease

Duchenne muscular dystrophy (DMD) is one of the rare fatal inherited muscular diseases, most patients with the disease are male (Crisafulli et al., 2020). Patients with DMD often die in their teens. Respiratory and cardiac support treatments can help extend period of life and survival, and physical therapy combined with hormone therapy can help improve quality level of life, but these treatments do not cure DMD (Kamdar and Garry, 2016; Mercuri et al., 2019). DMD is caused by the deletion of dystrophin due to a mutation in the DMD gene located on Xp21. The DMD gene is the largest known human gene (2.4Mb) and contains 79 exons, resulting in a diversity of types of mutations (Bladen et al., 2015). AAV gene therapy for the majority of DMD patients has been relatively safe in early clinical trials, and the transfer microdystrophin. gene using AAV has been shown to be widely effective in preclinical models (Verhaart and Aartsma-Rus, 2019). The therapy is highly transgenic and achieves stable high expression of dystrophin. In AAV gene therapy, high doses of AAV are required to treat the disease, and even if AAV is safe, it may cause an immune response (Colella et al., 2018). In conclusion, while early trials suggest AAV gene therapy may offer promise for Duchenne muscular dystrophy treatment, the approach still faces challenges, including potential immune responses.

##### 4.2.3.4 The application of AAV in cardiovascular diseases

Heart failure is common throughout the world, with the highest case fatality rate and hospitalization rate globally (Savarese and Lund, 2017). Because heart failure causes irreversible damage to the heart muscle, the use of drugs does not prevent the progression of heart failure. Heart failure patients need new treatments, and research has shown that cardiac gene therapy is a promising way to improve the contractility of patients’ heart muscles by targeting in regulation of sarcoendoplasmic reticulum ATPase (Zsebo et al., 2014) and phospholamban (Tsuji et al., 2009; Kairouz et al., 2012), protein phosphatase inhibitor1 correlating with beta-adrenergic system (Ishikawa et al., 2014; Watanabe et al., 2017). The transfection efficiency was provided by using AAV vector for gene therapy (Zacchigna et al., 2014). AAV-based gene therapy, targeting key cardiac regulators, shows promising potential as a novel approach in treating heart failure.

##### 4.2.3.5 The application of AAV in central nervous system disease

Parkinson’s disease is a progressive neurodegenerative disease characterized by tremors and motor retardation (Chen et al., 2020). Gene therapy is the one of the treatments for Parkinson’s disease. With the development of gene therapy for neurological diseases, AAV is increasingly used in the study of central nervous system diseases (Li and Samulski, 2020). With the iteration of gene therapy techniques, AAV capsid surface areas can be designed for different cell types and functions to cross the blood-brain barrier or retroactively transport through axons (Deverman et al., 2016; Tervo et al., 2016; Björklund and Davidsson, 2021). Based-AAV vector selectively deliver genes to nervous system cells for regulating its gene expression and tracing neural circuits, which promotes the research and application of neural circuits. Researchers studied the targeted projection of AAV genes into these neuronal regions in the substantia nigro and striatum to induce the production of neurotrophic factors to improve Parkinson’s disease (Albert et al., 2017; Kirik et al., 2017). The advantages of AAV vector gene therapy will have a broad application prospect for gene therapy of Parkinson’s disease by conducting more clinical trials.

#### 4.2.4 AAV and clinical drugs

Adeno-associated virus (AAV) vectors have been increasingly utilized in clinical research, demonstrating favorable safety profiles and notable clinical advantages across treating a spectrum of genetic diseases. In 2017, Luxturna®, employing the AAV2 vector, made history as the initial virus-based drug to receive approval from the U.S. Food and Drug Administration (FDA). Luxturna intends to address hereditary visual loss resulting from Leber congenital amaurosis or retinitis pigmentosa linked to precise genetic mutations. A pivotal Phase III clinical trial demonstrated a substantial visual improvement in a twelve-month period among patients identified with biallelic RPE65 gene mutations associated with retinal dystrophy, subsequent to Luxturna therapy (Russell et al., 2017). In 2019, Zolgensma®, also identified as AVXS-101 and utilizing the AAV9 vector, secured approval from the FDA for the treatment of Spinal Muscular Atrophy (SMA), constituting the second AAV gene therapy to garner such regulatory endorsement. SMA, predominantly occurring in childhood featured with the gradual degeneration of spinal motor neurons, arises from the deletion or mutation of the Survival Motor Neuron 1 (SMN1) gene (Lunn and Wang, 2008). The clinical trial START recruited 15 infants afflicted with infantile-onset SMA who underwent Zolgensma treatment, with all infants survival throughout the 24-month post-treatment follow-up period (Al-Zaidy et al., 2019). Hemgenix® (etranacogene dezaparvovec) represents a gene therapy utilizing the AAV5 vector to address Hemophilia B, expressing a hyperactive factor IX (FIX) transgene (FIX-padua). In 2022, Hemgenix obtained approval in the United States for Hemophilia B treatment. Roctavian® (valoctocogene roxaparvovec) utilizes an AAV5 vector to express complementary DNA for the B-domain-deleted factor VIII (FVIII). Approval has been granted by both the European Medicines Agency(EMA) and FDA for the treatment of severe Hemophilia A. Based on Phase III clinical trials, a substantial increase in FVIII or FIX levels was observed in the majority of treated patients (Mahlangu et al., 2023; Pipe et al., 2023). However, it is notable that the activity and expression of FVIII decrease over time. These issues require further resolution, particularly with regard to the persistence of FVIII expression and the substantial variability observed among different patients (Samelson-Jones and George, 2023). In 2023, ELEVIDYS® (delandistrogeny moxeparvovec) achieved the distinction of being the inaugural gene therapy sanctioned in the United States for the treatment of Duchenne muscular dystrophy (DMD). This therapeutic approach is applied to children between the ages of 4 and 5 who are diagnosed with DMD and possess mutations in the dystrophin (DMD) gene associated with deficient muscle dystrophin (Hoy, 2023).

While significant progress has been made in the development of the aforementioned drugs, the current use of first-generation AAV vectors requires relatively large vector doses to achieve clinical efficacy in the human body. The use of high doses has been demonstrated to elicit host immune responses, ultimately leading to severe adverse events. Recent incidents resulted in the unfortunate deaths of 10 patients (Wilson and Flotte, 2020; Guillou et al., 2022; Srivastava, 2023). Other complications include thrombocytopenia, renal failure, cardiopulmonary dysfunction, acute kidney injury, and atypical hemolytic-uremic syndrome, such as complement activation and platelet reduction (Srivastava, 2023). The prospect of developing a new generation of AAV vectors aims to achieve clinical efficacy with significantly reduced doses, thereby enhancing safety while simultaneously lowering economic costs.

## 5 Conclusion

In conclusion, gene therapy research related to AAV vectors has shown significant growth since 1991, with active global collaboration. The United States stands as the global leader in this field, with the University of Florida being the most prolific research institution. Wilson, James M emerges as a prominent figure with the highest number of published papers and co-citations. The study identified “gene editing,” “structure,” “CRISPR,” and “AAV application in inherent diseases” as hot topics in the field. Therefore, future research should focus on the application and development of AAV vectors in gene therapies for inherited diseases and explore ways to mitigate the negative effects of genetic diseases across various systems to advance human health. The comprehensive bibliometric analysis and visualization maps presented in this study provide valuable insights and serve as a useful reference for further research in this domain. By building upon these findings, researchers can continue to advance the field of gene therapy using AAV vectors, ultimately contributing to the betterment of human health.

## Data availability statement

The datasets presented in this study can be found in online repositories. The names of the repository/repositories and accession number(s) can be found in the article/**Supplementary Material**.

## Author contributions

FJ: Formal analysis, Visualization, Writing – original draft, Investigation, Software. CZ: Data curation, Formal analysis, Visualization, Writing – original draft, Software. WL: Formal analysis, Visualization, Writing – original draft, Investigation. FL: Formal analysis, Visualization, Writing – original draft. HH: Data curation, Software, Writing – review & editing. YT: Conceptualization, Software, Supervision, Visualization, Writing – review & editing. BQ: Funding acquisition, Supervision, Writing – review & editing.
