# Supplementary material for: Bibliometric analysis of global research trends in adeno-associated virus vector for gene therapy (1991-2022)
Source: Front Cell Infect Microbiol. 2023 Dec 8;13:1301915. doi: 10.3389/fcimb.2023.1301915 (PMC10739348; doi:10.3389/fcimb.2023.1301915)
Supplement: Supplementary file 1 [file Table_1.docx]

Supplementary Table 1. The top 20 most co-cited references related to AAV gene therapy.

| Rank | Title | DOI | Year | First Author | Journal | Local Citations (LC) | Global Citations (GC) | LC/GC Ratio (%) | Normalized Local Citations | Normalized Global Citations | Type of Article |
| --- | --- | --- | --- | --- | --- | --- | --- | --- | --- | --- | --- |
| 1 | Novel adeno-associated viruses from rhesus monkeys as vectors for human gene therapy | 10.1073/pnas.182412299 | 2002 | Gao Gp | Proceedings of the National Academy of Sciences of the United States of America | 638 | 1158 | 55.09 | 24.20 | 14.17 | article |
| 2 | Adenovirus-associated virus vector-mediated gene transfer in hemophilia B | 10.1056/NEJMoa1108046 | 2011 | Nathwani AC | New England Journal of Medicine | 552 | 1264 | 43.67 | 31.92 | 23.02 | article |
| 3 | Production of high-titer recombinant adeno-associated virus vectors in the absence of helper adenovirus | 10.1128/JVI.72.3.2224-2232.1998 | 1998 | Xiao X | Journal of Virology | 516 | 1074 | 48.04 | 11.97 | 9.07 | article |
| 4 | Safety and efficacy of gene transfer for Leber's congenital amaurosis | 10.1056/NEJMoa0802315 | 2008 | Maguire AM | New England Journal of Medicine | 487 | 1599 | 30.46 | 18.98 | 19.71 | article |
| 5 | Membrane-associated heparan sulfate proteoglycan is a receptor for adeno-associated virus type 2 virions | 10.1128/JVI.72.2.1438-1445.1998 | 1998 | SummerfordC | Journal of Virology | 483 | 1047 | 46.13 | 11.20 | 8.84 | article |
| 6 | Clades of Adeno-associated viruses are widely disseminated in human tissues | 10.1128/JVI.78.12.6381-6388.2004 | 2004 | Gao Gp | Journal of Virology | 431 | 707 | 60.96 | 22.28 | 10.48 | article |
| 7 | Effect of gene therapy on visual function in Leber's congenital amaurosis | 10.1056/NEJMoa0802268 | 2008 | Bainberidge JWB | New England Journal of Medicine | 414 | 1485 | 27.88 | 16.14 | 18.30 | article |
| 8 | Long-term safety and efficacy of factor IX gene therapy in hemophilia B | 10.1056/NEJMoa1407309 | 2014 | Nathwani AC | New England Journal of Medicine | 397 | 823 | 48.24 | 27.53 | 17.23 | article |
| 9 | Recombinant adeno-associated virus purification using novel methods improves infectious titer and yield | 10.1038/sj.gt.3300938 | 1999 | Zolotukhin S | Gene Therapy | 395 | 985 | 40.10 | 10.97 | 10.83 | article |
| 10 | Prevalence of serum IgG and neutralizing factors against adeno-associated virus (AAV) types 1, 2, 5, 6, 8, and 9 in the healthy population: implications for gene therapy using AAV vectors | 10.1089/hum.2009.182 | 2010 | Boutin S | Gene Therapy | 339 | 581 | 58.35 | 18.22 | 10.04 | article |
| 11 | Worldwide epidemiology of neutralizing antibodies to adeno-associated viruses | 10.1086/595830 | 2009 | Calcedo R | The Journal of Infectious Diseases | 325 | 502 | 64.74 | 17.53 | 8.13 | article |
| 12 | Long-term gene expression and phenotypic correction using adeno-associated virus vectors in the mammalian brain | 10.1038/ng1094-148 | 1994 | Kaplitt MG | Nature Genetics | 320 | 868 | 36.87 | 4.88 | 4.76 | article |
| 13 | Cross-packaging of a single adeno-associated virus (AAV) type 2 vector genome into multiple AAV serotypes enables transduction with broad specificity | 10.1128/JVI.76.2.791-801.2002 | 2002 | Rabinowitz JE | Journal of Virology | 319 | 519 | 53.98 | 12.10 | 7.23 | article |
| 14 | Adeno-associated virus serotypes: vector toolkit for human gene therapy | 10.1016/j.ymthe.2006.05.009 | 2006 | Wu ZJ | Molecular Therapy | 293 | 570 | 51.40 | 12.87 | 8.70 | review |
| 15 | Second-strand synthesis is a rate-limiting step for efficient transduction by recombinant adeno-associated virus vectors | 10.1128/JVI.70.5.3227-3234.1996 | 1996 | Ferrari FK | Journal of virology | 290 | 576 | 50.35 | 5.66 | 4.58 | article |
| 16 | Human fibroblast growth factor receptor 1 is a co-receptor for infection by adeno-associated virus 2 | 10.1038/4758 | 1999 | Qing K | Nature Medicine | 279 | 522 | 53.45 | 7.75 | 5.74 | article |
| 17 | CD8(+) T-cell responses to adeno-associated virus capsid in humans | 10.1038/nm1549 | 2007 | Mingozzi F | Nature Medicine | 272 | 504 | 32.86 | 11.50 | 6.47 | article |
| 18 | Novel tools for production and purification of recombinant adenoassociated virus vectors | 10.1089/hum.1998.9.18-2745 | 1998 | Grimm D | Human Gene Therapy | 267 | 541 | 49.35 | 6.19 | 4.57 | article |
| 19 | Next generation of adeno-associated virus 2 vectors: point mutations in tyrosines lead to high-efficiency transduction at lower doses | 10.1073/pnas.0802866105 | 2008 | Zhong L | Proceedings of the National Academy of Sciences of the United States of America | 266 | 411 | 64.72 | 10.37 | 5.06 | article |
| 20 | Targeted integration of adeno-associated virus (AAV) into human chromosome 19 | 10.1002/j.1460-2075.1991.tb04964.x | 1991 | Samulski RJ | The EMBO Journal | 265 | 612 | 43.30 | 1.00 | 1.00 | article |

Supplementary Table 2. Reference co-occurrence network clustering table.

| Cluster ID | Size | Silhouette | Mean (year) | Top terms (LSI) | Top terms (LLR) | Top terms (MI) |
| --- | --- | --- | --- | --- | --- | --- |
| 0 | 69 | 0.945 | 2005 | gene therapy; factor viii | heart; vascular endothelial growth factor | adeno-associated virus 8; naked dna delivery |
| 1 | 66 | 0.924 | 1999 | gene therapy; adeno-associated virus | autoimmunity ; cd34(+) | receptor targeting; lipofection |
| 2 | 62 | 0.975 | 2017 | gene therapy; adeno-associated virus | duchenne muscular dystrophy; crispr | deamidation; development challenges |
| 3 | 57 | 0.954 | 2012 | gene therapy; hemophilia b; clinical trial | immune responses; receptor | correlation; large animal models |
| 4 | 53 | 0.912 | 1990 | gene therapy; adenovirus vectors | adenoassociated virus (aav); nonpathogenic | gene therapy; adeno-associated virus |
| 5 | 47 | 0.965 | 2011 | gene therapy; adeno-associated virus | retina ; rpe65 | non-viral gene therapy; lentiviral vector |
| 6 | 45 | 0.927 | 1993 | gene therapy; adenoassociated virus; cystic fibrosis | hematopoiesis; adenoassociated virus | vessel wall; lcr |
| 7 | 43 | 0.932 | 1997 | gene therapy; injection parameters | arthritis; erythropoietin | hemophilic dogs; liver stem cells |
| 8 | 37 | 0.992 | 1988 | aav; adenoassociated virus | chromosome; human | gene therapy; adeno-associated virus |
| 9 | 30 | 0.973 | 2010 | gene therapy; adeno-associated virus | central nervous system; intrathecal | non-human primates; perinatal |
| 10 | 30 | 0.981 | 1990 | … | receptor targeting; animal models | gene therapy; adeno-associated virus |
| 12 | 20 | 0.986 | 1992 | gene therapy; adeno-associated virus | liposomes; primary epithelial cell | gene therapy; adeno-associated virus |
| 13 | 7 | 1 | 1989 | ... | receptor targeting; animal models | gene therapy; adeno-associated virus |
| 14 | 6 | 0.997 | 1992 | ... | receptor targeting; animal models | gene therapy; adeno-associated virus |

Supplementary Table 3. Keywords co-occurrence network clustering table.

| Cluster ID | Size | Silhouette | Mean (year) | Top terms (LSI) | Top terms (LLR) | Top terms (MI) |
| --- | --- | --- | --- | --- | --- | --- |
| 0 | 40 | 0.789 | 1998 | gene therapy; cells | vectors ; expression | alzheimer 's disease ; centrosomal protein |
| 1 | 38 | 0.787 | 1994 | gene therapy; adeno-associated virus | adenoassociated virus; mammalian cells | compartment ; doxorubicin |
| 2 | 32 | 0.895 | 1994 | gene therapy; gene transfer | retrovirus vectors ; locus control region | gene therapy ; adeno-associated virus |
| 3 | 29 | 0.753 | 2004 | gene therapy; hemophilia b | factor ix; hemophilia b | a i; aav-mediated gene therapy |
| 4 | 29 | 0.862 | 1999 | gene therapy; viral vectors | adeno-associated virus; gene therapy | alzheimer 's disease ; centrosomal protein |
| 5 | 28 | 0.845 | 2000 | gene therapy; central nervous system | central nervous system ; mouse model | alzheimer 's disease ; centrosomal protein |
| 6 | 22 | 0.928 | 1994 | gene therapy; storage disease | vector; vectors | alzheimer 's disease; inflammatory bowel diseases |
| 7 | 15 | 0.985 | 1992 | adenoassociated virus; genome | inhibits cellular transformation; chromosome | gene therapy ; adeno-associated virus |
| 8 | 15 | 0.915 | 1994 | gene therapy; growth factor | erythroid cells; hematopoiesis | gene therapy ; adeno-associated virus |
| 9 | 12 | 0.827 | 2011 | gene therapy; adeno-associated virus | safety; leber congenital amaurosis | centrosomal protein; fix transgene |
| 11 | 5 | 0.984 | 1993 | human gene; adeno-associated virus | sequence; minute virus | gene therapy ; adeno-associated virus |

Supplementary Table 4. The top 10 most co-cited references related to AAV gene therapy in 2023.

| Rank | Title | DOI | Year | First Author | Journal | Local Citations (LC) | Global Citations (GC) | LC/GC Ratio (%) | Normalized Local Citations | Normalized Global Citations | Type of Article |
| --- | --- | --- | --- | --- | --- | --- | --- | --- | --- | --- | --- |
| 1 | Gene Therapy with Etranacogene Dezaparvovec for Hemophilia B | 10.1056/NEJMoa2211644 | 2023 | Pipe SW | New England Journal of Medicine | 6 | 21 | 28.57 | 23.50 | 20.29 | article |
| 2 | Adeno-Associated Virus Gene Therapy for Hemophilia | 10.1146/annurev-med-043021-033013 | 2023 | Samelson-Jones BJ | Annual Review of Medicine | 5 | 14 | 35.71 | 19.59 | 13.52 | review |
| 3 | Biodistribution of Adeno-Associated Virus Gene Therapy Following Cerebrospinal Fluid-Directed Administration | 10.1089/hum.2022.163 | 2023 | Chen X | Human Gene Therapy | 5 | 7 | 71.43 | 19.59 | 6.76 | review |
| 4 | Genomic investigations of unexplained acute hepatitis in children | 10.1038/s41586-023-06003-w | 2023 | Morfopoulou S | Nature | 4 | 16 | 25.00 | 15.67 | 15.46 | article |
| 5 | Two-Year Outcomes of Valoctocogene Roxaparvovec Therapy for Hemophilia A | 10.1056/NEJMoa2211075 | 2023 | Mahlangu J | New England Journal of Medicine | 4 | 17 | 25.23 | 15.67 | 16.42 | article |
| 6 | Various AAV Serotypes and Their Applications in Gene Therapy: An Overview | 10.3390/cells12050785 | 2023 | Issa SS | Cells | 3 | 7 | 42.86 | 11.75 | 6.76 | review |
| 7 | Neonatal Fc Receptor Inhibition Enables Adeno-Associated Virus Gene Therapy Despite Pre-Existing Humoral Immunity | 10.1089/hum.2022.216 | 2023 | Horiuchi M | Human Gene Therapy | 3 | 3 | 100.00 | 11.75 | 2.90 | article |
| 8 | AAV vectors applied to the treatment of CNS disorders: Clinical status and challenges | 10.1016/j.jconrel.2023.01.067 | 2023 | Kang Lin | Journal of Controlled Release | 3 | 6 | 50.00 | 11.75 | 5.80 | review |
| 9 | Immune transgene-dependent myocarditis in macaques after systemic administration of adeno-associated virus expressing human acid alpha-glucosidase | 10.3389/fimmu.2023.1094279 | 2023 | Hordeaux J | Frontiers in Immunology | 3 | 3 | 100.00 | 11.75 | 2.90 | article |
| 10 | Etranacogene Dezaparvovec: First Approval | 10.1007/s40265-023-01845-0 | 2023 | Heo YA | Drugs | 3 | 8 | 37.50 | 11.75 | 7.73 | review |
